# Supplementary material for: Validation of a Microfluidic Device Prototype for Cancer Detection and Identification: Circulating Tumor Cells Classification Based on Cell Trajectory Analysis Leveraging Cell-Based Modeling and Machine Learning
Source: bioRxiv. 2024 Aug 20:2024.08.19.608572. Preprint. [Version 1] doi: 10.1101/2024.08.19.608572 (PMC11370430; doi:10.1101/2024.08.19.608572)
Supplement: 1 [file NIHPP2024.08.19.608572V1-supplement-1.pdf]

bioRxiv preprint doi: https://doi.org/10.1101/2024.08.19.608572; this version posted August 20, 2024. The copyright holder for this preprint (which was not certified by peer review) is the author/funder, who has granted bioRxiv a license to display the preprint in perpetuity. It is made available under aCC-BY 4.0 International license.

Supporting information

S1 Code Trajectory data and code The cell trajectory data and machine learning code can be downloaded from
https://github.com/imsanjoykb/vMDpcDI-CTC_Modeling.git

S2 Code Cell based simulation code The implementation and settings of our cancer cell simulation based on the Hemocell library [19] can be found here.
https://github.com/qcutexu/CellBasedModeling.git

S3 Video Case (A) A movie showing the RBC tank treading motion under the shear rate $\dot{\gamma} = 100s^{-1}$.

S4 Video. Case (B) A movie showing the RBC tank treading motion under the shear rate $\dot{\gamma} = 500s^{-1}$.

S5 Video. Case (B) A movie showing a mixed tank treading and tumbling motions under the shear rate $\dot{\gamma} = 250s^{-1}$.

bioRxiv preprint doi: https://doi.org/10.1101/2024.08.19.608572; this version posted August 20, 2024. The copyright holder for this preprint (which was not certified by peer review) is the author/funder, who has granted bioRxiv a license to display the preprint in perpetuity. It is made available under aCC-BY 4.0 International license.

Layer (type)	Output Shape	Param #	Connected to
Input Layer 1	(559, 3)	0	-
Input Layer 2	(559, 3)	0	-
Convolution1D 11	(557, 64)	640	Input Layer 1
Convolution1D 21	(557, 64)	640	Input Layer 2
MaxPooling1D 11	(278, 64)	0	Convolution1D 11
MaxPooling1D 21	(278, 64)	0	Convolution1D 21
Convolution1D 12	(276, 64)	12,352	MaxPooling1D 11
Convolution1D 22	(276, 64)	12,352	MaxPooling1D 21
GlobalAveragePooling1D 1	64	0	Convolution1D 12
GlobalAveragePooling1D 2	64	0	Convolution1D 22
Concatenate	128	0	GlobalAveragePooling1D 1<br>GlobalAveragePooling1D 2
Dense 1	128	16,512	Concatenate
Dense 2	64	8,256	dense 1
Dense 3	2	130	dense 2

S6 Table. The configuration of RNN The details of the recurrent layers and hidden layers are provided in the table below.

Layer (type)	Output Shape	Param #
Bidirectional 1	(None, 559,256)	104,448
Batch normalization 1	(None, 559, 256)	1,024
Dropout 1	(None, 559,256)	0
Bidirectional 2	(None, 559,128)	123,648
Batch normalization 2	(None, 559,128)	512
Dropout 2	(None, 559,128)	0
Bidirectional 3	(None, 64)	31,104
Batch normalization 3	(None, 64)	256
Dropout 3	(None, 64)	0
Dense 1	(None, 16)	1,040
Dense 2	(None, 2)	34

S7 Table. The configuration of CNN The details of the convolutional layers and hidden layers are provided in the table below.
